# Silica exposure and work-relatedness evaluation for occupational cancer in Korea

**DOI:** 10.1186/s40557-018-0216-1

**Published:** 2018-01-31

**Authors:** Hyoung-Ryoul Kim, Boowook Kim, Bum Seak Jo, Ji-Won Lee

**Affiliations:** 1 Department of Occupational and Environmental Medicine, College of Medicine, The Catholic University of Korea, 222 Banpo-Daero, Seocho-Gu, Seoul, 137701 Republic of Korea; 2 Occupational Lung Disease Institute, Korea Workers’ Compensation and Welfare Service, Incheon, Korea

**Keywords:** Crystalline silica, Lung cancer, Pneumoconiosis

## Abstract

Crystalline silica has been classified as a definite carcinogen (Group 1) causing lung cancer by the International Agency for Research on Cancer (IARC). In Korea, crystalline silica has been the most common causal agent for workers to apply to the Korea Workers’ Compensation and Welfare Service (KWCWS). We used KWCWS data to evaluate workers’ crystalline silica exposure levels according to their occupations and industries, and reviewed research papers describing the dose-response relationship between cumulative exposure levels and lung cancer incidence. In addition, we reviewed lung cancer cases accepted by the KWCWS, and suggest new criteria for defining occupational cancer caused by crystalline silica in Korea. Rather than confining to miners, we propose recognizing occupational lung cancer whenever workers with pneumoconiosis develop lung cancer, regardless of their industry. Simultaneous exposure and lag time should also be considered in evaluations of work-relatedness.

## Background

Silica, or silicon dioxide (SiO2), is a group IV metal oxide, which naturally occurs in both crystalline and amorphous forms. The most abundant form of silica is α-quartz, and the term quartz is often used in place of the general term crystalline silica. Crystalline silica has been classified as a definite carcinogen (Group 1) causing lung cancer by the International Agency for Research on Cancer (IARC) [1]. In some studies, kidney, stomach, and esophageal cancers have also been reported to be associated with crystalline silica [2, 3]. In Korea, mining, stone cutting, construction, and refining are known to be hazardous industries with respect to crystalline silica exposure, and many silicosis cases have occurred among these industries [4]. There has been no systematic survey on the number of workers exposed to crystalline silica in Korea, but in one survey in 2005, the number of workers handling crystalline silica was estimated to be about 48,000, while the number of crystalline silica manufacturing work sites was estimated to be 190 [5].

At present, when workers with silicosis (or pneumoconiosis) over stage 1/0 who have worked as miners are diagnosed with lung cancer, it is recognized as occupational lung cancer without further investigation through Act on the prevention of pneumoconiosis and protection, Etc. of workers suffering from pneumoconiosis. However, this operational definition is not applied for manufacturing workers, so most lung cancer cases, even in individuals with silicosis, are investigated by the KWCWS. Lung cancer cases who do not have silicosis, but have long working histories in mines are also investigated for defining occupational cancer.

The IARC has defined crystalline silica as a definite carcinogen inducing lung cancer, but not other cancers (kidney, stomach, laryngeal and esophageal cancer) [1]. Until now, only a few studies have evaluated the exact cumulative exposure level that induces lung cancer.

Therefore, for this article, we first used KWCWS data to evaluate crystalline silica exposure levels in Korean workers according to their occupations and industries. Second, we reviewed IARC report, National Institute of Occupational Safety and Health (NIOSH) report, UK Industry Injury Advisory Committee (IIAC) report and American Conference of Governmental Industrial Hygienists (ACGIH) documentation describing crystalline silica. Third, we reviewed research papers describing the dose-response relationship between cumulative crystalline silica exposure levels and lung cancer incidence. Fourth, we reviewed lung cancer cases accepted by the KWCWS. Fifth, we suggested new criteria for defining occupational cancer caused by crystalline silica in Korea.

## Reviews

### Occupational exposure to crystalline silica in Korea

In many industries, workers are exposed to crystalline silica and experience occupational diseases such as lung cancer and silicosis; however, workers are often unaware of the health hazards of crystalline silica. In addition, government management has been insufficient because silica is not a major component of most processes. However, silica exposure still occurs as a byproduct. The ACGIH TLV-TWA level of silica is 0.025 mg/m^3^, while the Korean OEL (Occupational Exposure Limit) is 0.05 mg/m^3^. In some occupations, exposure levels exceed the Korean OEL. Further, in industries such as construction and stone cutting, most workers are temporary workers and are thus outside the occupational health management system (Table 1).

**Table 1:** Crystalline silica exposure levels investigated by the KWCWS

Industry	Exposure level^a^	Concentration (mg/m^3^)
Mining	High/Medium	0.054 (rock drilling) 0.027 (coal face mining)
Foundry work	High/Medium	0.028 0.233 (factory using sand sprayer)
Construction	High/Medium	0.399 (concrete grinding) 0.042 (stone laying work)
Stone quarrying	High	0.060
Stone processing	High	0.796 (dry process) 0.067 (wet process)
Ceramics	Medium	0.028 (diatomaceous earth)
Briquetting	Medium	0.048
Calcium carbonate manufacturing	Medium	0.038
Paint manufacturing	Low	0.007
Horse racetrack (horse training)	Low	0.018
Subway work	Low	0.006
Agriculture	Low	0.014 (potato cultivation)

^a^High (> 0.05 mg/m^3^), medium (0.025 < and < 0.05 mg/m^3^) Low (< 0.025 mg/m^3^)

### IARC report (2012) [1]

An IARC report published in 1997 supported the increased risk of lung cancer in patients with silicosis. 1) When adjustment was made for the effects of smoking, the risk of lung cancer (RR or SMR) in patients with silicosis was more than double that in the non-silicosis-exposed individuals or the general population. 2) An exposure-response relationship was observed in various studies. 3) Consistent results about risk have been obtained across different countries, industries and time periods. 4) Amandus et al. [6–8] and Partanen et al. [9] provided valid evidence for the association. Tsuda et al. [10] conducted a meta-analysis of lung cancer risk in workers with silicosis and pneumoconiosis, while excluding those with asbestosis. The pooled risk was 2.74 (95% CI 2.60–2.90) in 32 mortality studies published from 1980 to 1994, and the rate ratio was 2.77 (95% CI 2.61–2.94), while the rate ratio was 2.84 (95% CI 2.25–3.59) when only a case-control study was performed.

The strongest evidences supporting the carcinogenicity of crystalline silica in the lungs are from pooled and meta-analyses. Cancers other than that of the lung have not been researched as thoroughly. In many cases, the findings have been reported focused on lung cancer. In examinations of the relationship between crystalline silica and lung cancer, it is appropriate to evaluate the diatomaceous earth and sand industries, where there are minimal confounding variables (radon and other sources of ionizing radiation). Studies in these industries have consistently demonstrated the association between crystalline silica exposure and lung cancer incidence. In a situation where it was difficult to completely exclude the role of confounding variables, a meta-analysis was conducted to verify the carcinogenicity of crystalline silica. These results confirmed that crystalline silica is a definite carcinogen inducing lung cancer. However, other cancers have not been studied sufficiently thus far.

### NIOSH report (2002) [11]

In a recent case-control study in Canada, Parent et al. [12] found that 25 of 250 pathologically confirmed gastric cancer patients had significant levels of occupational exposure to crystalline silica. The odds ratio (OR) was 1.7 (95% CI = 1.1–2.7) in these patients compared with 2822 control subjects after adjustment for age, place of birth, education level and smoking.

On the other hand, Cocco et al. [13] reviewed epidemiological studies on the association between stomach cancer and dust exposure. They found that adjustment for confounding variables was not performed in most studies, and concluded that crystalline silica was not a sufficient carcinogen to induce gastric cancer. Other cancers such as nasopharyngeal cancer, salivary gland cancer, liver cancer, bone cancer, pancreatic cancer, skin cancer, esophageal cancer, digestive cancer, intestinal or peritoneal cancer, lymphatic hematopoietic cancer, brain cancer and bladder cancer have not been established as cancers caused by crystalline silica. The NIOSH perspective was similar to that of the IARC.

### UK IIAC report (2010) [14]

In the UK IIAC report issued in 2010, there was no change in the principle of providing compensation for workers with silicosis who develop lung cancer. These criteria had been maintained since 1992, when the IIAC recognized lung cancer as an occupational injury only in workers with silicosis, based on a two-fold increase in risk. However, in the absence of silicosis, the association with lung cancer was not acknowledged. The term of prescription was limited to patients with silicosis who had been employed in one of the following 10 industries: glass manufacturing, sandstone tunneling and quarrying, pottery, metal ore mining, slate quarrying and production, clay mining, using siliceous materials as abrasives, foundry work, granite tunneling and quarrying, and stone cutting and masonry.

In 1997, the IARC defined crystalline silica as a Group 1 definite carcinogen associated with lung cancer. However, most reviews only included subjects with silicosis, and no formal meta-analysis was conducted. In this review, there was a significant increase in lung cancer risk in 84% of the studies conducted. However, these studies did not reveal significant increases in all industries. In addition, many studies failed to exclude strong confounding variables such as smoking.

In 2006, Pelucchi et al. [15] summarized 35 studies (cohort studies and case-control studies) of subjects without silicosis, and found that occupational silica exposure increased the risk of developing lung cancer in most studies. However, only one of these studies (a tin mining study in China) reported an increase in risk greater than two-fold. In 11 cohorts of patients with silicosis, the mRR was 1.69 (95% confidence interval 1.32–2.16), and three studies revealed an increase in risk greater than 2-fold. A number of studies have been published since 2006, but the IIAC has not found any clear evidence warranting changes to the conclusions of 1992.

### ACGIH documentation (2010) [16]

In an epidemiological study in 2001, Steenland et al. found an increased risk of lung cancer in the group with an average crystalline silica concentration ≥ 0.065 mg/m^3^ (reference: 0–0.023 mg/m^3^) [17]. On the basis of this study, the TLV-TWA of crystalline silica was considered to be too high, so the 8-h weighted average was changed to 0.025 mg/m^3^. The mean exposure period for this group was 8.8 years. In this study, the cumulative dose of crystalline silica with an increase of a significant SMR was 1.28 mg/m^3^-year.

### Review of recognized occupational lung cancers in Korea

Since 2007, we have analyzed 120 occupational lung cancer cases approved by the KWCWS. All the affected individuals were male, and 14.2% had no smoking history, while 37.5% had a history of mine work. After mining, the next most common occupation was stone processing, including cutting, grinding, construction and foundry work. Numerous cases were approved among painters and subway workers. There were four cases of horse trainers working at a racetrack, and calcium carbonate manufacturing workers were also approved for crystalline silica-induced lung cancer. Most workers were exposed to multiple carcinogens (72.5%). Major coincidental exposure with silica included asbestos, diesel exhaust, radon and hexavalent chromium. From the carcinogenic exposure to the onset of the disease, there was no case of less than 10 years, and lag times of more than 40 years were reported in 40% of the cases. Of the recognized cases, 19.2% had pneumoconiosis.

Most of the miners were engaged in coal mining and excavation. The work duration was more than 10 years in most cases, but three cases were approved for occupational disease designation with work duration of less than 10 years (Table 2).

**Table 2:** Characteristics of occupational lung cancer cases recognized by the KWCWS

Variables	Classification	Frequency (%)
Age	Less than 40 years	2 (1.7)
	40–49 years	9 (7.5)
	50–59 years	36 (30.0)
	60–69 years	35 (29.2)
	More than 70 years	38 (31.7)
Sex	Male	120 (100)
	Female	0 (0)
Smoking history	None	17 (14.2)
	Less than 20PY	33 (27.5)
	More than 20PY	63 (52.5)
	Unknown	7 (5.8)
Industries	Mining	45 (37.5)
	Stone quarrying, processing	13 (10.8)
	Foundry work	10 (8.3)
	Construction	13 (10.8)
	Subway work	8 (6.7)
	Painting or paint manufacturing	9 (7.5)
	Horse training	4 (3.3)
	Briquetting	4 (3.3)
	Car repair	2 (1.7)
	Fireproof material work	2 (1.7)
	Others	10 (8.3)
Pneumoconiosis	Yes	23 (19.2)
	No	97 (80.2)
Lag time	Less than 10 years	0 (0)
	10–19 years	15 (12.5)
	20–29 years	20 (16.7)
	30–39 years	37 (30.8)
	More than 40 years	48 (40.0)

## Discussion

The main considerations in work-relatedness applications and determinations were the presence of pneumoconiosis, cumulative exposure estimates, coincidental exposures, associations with other cancers, and domestic exposure levels, and current domestic approval trends.

### The relationship between silica exposure and lung cancer according to the presence or absence of pneumoconiosis

Steenland et al. published an integrated analysis of 10 cohorts in 2001 [17]. In this study, crystalline silica exposure was significantly associated with lung cancer incidence. Numerous papers have described the relationship between silicosis and lung cancer. The IARC considers the presence or absence of silicosis to be a biomarker for high crystalline silica exposure levels [1].

Checkoway et al. analyzed the risk of lung cancer using death data from workers in industries related to diatomaceous earth from 1987 to 1994. In this 1999 report, the standardized mortality ratio (SMR) for lung cancer was 1.57 (95% CI = 0.43–4.03) in workers with silicosis and 1.19 (95% CI = 0.87–1.57) in workers without silicosis [18]. The SMR was calculated according to the expected number of deaths among white males in the US, adjusted for age and calendar year. A statistically significant (*p* = 0.02) dose-response relationship was demonstrated between the SMR for lung cancer and the cumulative exposure to crystalline silica in workers without silicosis. The SMRs of lung cancer were 1.05 (95% CI = 0.56–1.9) for exposures less than 0.5 mg/m^3^-year, 0.86 (95% CI = 0.46–1.48) for 0.5–1.9 mg/m^3^-year, 1.25 (95% CI = 0.60–2.29) for 2.0–4.9 mg/m^3^-year, and 2.40 (95% CI = 1.24–4.20) for greater than 5 mg/m^3^-year. These results illustrate that silicosis may not be a necessary step in the course of lung cancer due to crystalline silica exposure.

Liu et al. published a long-term cohort study of 34,000 workers in China. Separate analysis of workers without silicosis revealed that a cumulative exposure ≥1.12 mg/m^3^-year increased the risk of lung cancer mortality (hazard ratio 1.42, 95% CI 1.06–1.92) [19]. Although many studies have demonstrated that silicosis potently induces the pathogenesis of lung cancer, recent studies using major cohorts have indicated that lung cancer is highly associated with cumulative crystalline silica exposure, even without silicosis. The IARC defines crystalline silica itself as a definite carcinogen, not silicosis (Group I).

### Other cancers besides lung cancer

Kidney cancer, stomach cancer, and esophageal cancer due to crystalline silica have been reported in various epidemiological studies. However, it is difficult to say that crystalline silica increases the incidence of cancers besides lung cancer, because the results of many epidemiological studies have not been consistent, and there has not been full adjustment for confounding variables.

### Cumulative exposure level related to lung cancer

Several studies have estimated the cumulative exposure dose of crystalline silica (with or without silicosis) that is associated with lung cancer development. The cumulative exposure dose has varied from 0.01 mg/m^3^-year [20] to 5 mg/m^3^-year [18]. In a 2001 study conducted in the United States by Steenland et al., the SMR among 18 sand mill workers compared with the general population was 2.25 (95% CI 1.48–3.27). At this point, the cumulative exposure level was greater than 1.28 mg/m^3^-year. This study became a major reference in the IARC report, and was cited by ACGIH as a basis for lowering the TLV-TWA to 0.025 mg/m^3^ [17].

In recent years, however, some reports have suggested other cumulative exposure levels associated with lung cancer incidence or mortality, such as 0.026 mg/m^3^-year [21] and 1.12 mg/m^3^-year [19]. In previous researches, cumulative exposure levels inducing lung cancer vary. So, it is a conflicting issue to define definite cumulative exposure level which can induce lung cancer.

### Occupational exposure level to silica in Korea

Crystalline silica exposure levels have been evaluated in several industries. A high level of exposure was found in the construction industry, and significant exposures were also confirmed among miners and foundry workers. Stone quarrying and stone laying were also found to expose workers to high concentrations of crystalline silica. When the dry method of stone quarrying was used, the exposure level was extremely high. Thus, it appeared that the crystalline silica exposure level was the highest for stone quarrying, followed by stone laying and mining.

### Simultaneous exposures

In general, industries in which workers are exposed only to crystalline silica are uncommon. In mines, workers are exposed simultaneously to crystalline silica, diesel exhaust particles and radon, and the effect of concurrent exposure to these carcinogens is as strong as the effect of crystalline silica exposure. In cases involving multiple exposures, it is not appropriate to set the acceptance criteria based on the cumulative exposure to crystalline silica alone. Rather, the magnitude of risk due to each coexisting carcinogen must be considered at the same time, and even if there is a relatively low level of crystalline silica exposure, the case should be reviewed for approval of work-relatedness. In particular, it is necessary to determine whether there has been simultaneous exposure to asbestos, diesel exhaust particles, radon, hexavalent chromium and poly-aromatic hydrocarbons.

### Current domestic approval trends

A review of 120 cases of occupational lung cancer confirmed through an epidemiological survey of the KWCWS since 2007 revealed that 45 cases (37.5%) involved miners. Stone quarrying, construction and foundry work followed. Numerous cases were approved among painters and subway workers. Characteristically, there were four cases of horse trainers working at a racetrack, and some cases were approved for workers in phosphorus stone treatment and calcium carbonate manufacturing. Most of the coincidental exposures (72.5%) involved asbestos, diesel exhaust particles, radon, hexavalent chromium, and so on. From the carcinogenic exposure to the onset of the disease, there was no case of less than 10 years, and the lag time was more than 40 years in 40% of the cases.

Among the patients whose cases were approved, 19.2% had pneumoconiosis, while many cases were approved without pneumoconiosis. This is because when a patient has both pneumoconiosis and a history of mine work, lung cancer is automatically recognized, and many such cases do not go through epidemiological investigation. Nonetheless, there were many cases in industries other than mining that were approved for occupational lung cancer designation without silicosis.

## Conclusions

The authors suggest the following guidelines for recognizing occupational cancer due to crystalline silica. At present, lung cancer is recognized as a complication in patients with pneumoconiosis (above stage 1/0) only when they have a history of mine work. However, it is not appropriate to confine mining work. Rather, when workers with pneumoconiosis (above stage 1/0) develop lung cancer, it should be recognized as occupational lung cancer, regardless of the industries in which they are engaged. In the absence of silicosis, the work-relatedness of lung cancer should be judged considering the cumulative exposure level, simultaneous exposure, working period, and lag time.
